# Hyperammonemic encephalopathy requiring hemodialysis in a child with distal renal tubular acidosis

**DOI:** 10.1007/s40620-025-02218-4

**Published:** 2025-02-07

**Authors:** Behruz Huseynli, Emine Akkuzu, Bahar Büyükkaragöz, Sevcan A. Bakkaloğlu

**Affiliations:** 1 Department of Pediatric Nephrology, Gazi University, Besevler, 06560 Ankara, Turkey; 2 Pediatric Intensive Care Unit, Gazi University, Ankara, Turkey

**Keywords:** Distal renal tubular acidosis, Hyperammonemia, Hyperammonemic encephalopathy, Hemodialysis

## Abstract

A 3.5-year-old girl with genetically proven distal renal tubular acidosis presented with lethargy, after numerous episodes of vomiting and poor feeding. Laboratory investigations revealed severe metabolic acidosis, hypokalemia and a serum ammonia level of 515 mmol/L (normal range: 19–50 mmol/L). Despite treatment with sodium bicarbonate, potassium supplementation, sodium benzoate and carglumic acid, her condition required hemodialysis, which resulted in rapid improvement in clinical and metabolic parameters. Hyperammonemia in distal renal tubular acidosis results from impaired ammonium excretion and increased ammoniagenesis due to hypokalemia and chronic metabolic acidosis, particularly during metabolic decompensation. This case had the highest ever reported serum ammonia level in distal renal tubular acidosis with encephalopathic findings, necessitating hemodialysis treatment. Routine monitoring of serum ammonia levels in distal renal tubular acidosis patients during metabolic stress is essential.

**Graphical Abstract:**

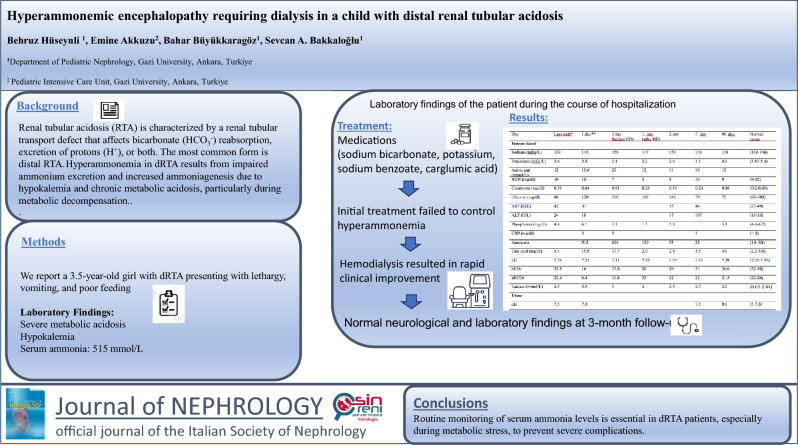

## Introduction

Renal tubular acidosis (RTA) is characterized by a renal tubular transport defect that affects bicarbonate (HCO_3_^−^) reabsorption, excretion of protons (H^+^), or both. The disease can be classified into three major categories as distal renal tubular acidosis ([dRTA] or type 1 renal tubular acidosis), proximal renal tubular acidosis (type 2 renal tubular acidosis) and type 4 renal tubular acidosis. The most commonly observed form is distal renal tubular acidosis, which is caused by a defect in distal tubular acidification, specifically an inability to secrete H^+^ ions in the type A intercalated cells of the collecting tubules. Distal renal tubular acidosis is usually primary (genetic) in children and is associated with mutations in five genes: *ATP6V0A4, ATP6V1B1, SLC4A1, FOXI1,* and *WDR72*. It may also develop secondary to systemic pathologies, drug intake or exposure to toxic substances [1, 2]. The disease is characterized by normal anion gap metabolic acidosis accompanied by hypokalemia, hypercalciuria, hypocitraturia, or nephrocalcinosis. Failure to reduce urine pH below 5.3–5.5, along with a positive urine anion gap despite metabolic acidosis, also serve as diagnostic indicators. Failure to thrive, polyuria, polydipsia, diarrhea, vomiting, and rickets are common findings in children with distal renal tubular acidosis. Furthermore, the metabolism of ammonia may be impaired in distal renal tubular acidosis leading to hyperammonemia in children. Aside from reduced ammonia excretion in these patients, the presence of metabolic acidosis and hypokalemia increase renal ammonia synthesis [3–8]. In this article, we report the highest ever reported serum ammonia level in a child with distal renal tubular acidosis causing hyperammonemic encephalopathy. Unlike previous cases, this patient required hemodialysis for hyperammonemic encephalopathy and deep metabolic acidosis.

## Case report

A three-and-a-half-year-old girl was brought to the emergency room after over ten episodes of non-projectile vomiting, poor feeding in the prior two days, and onset of lethargy in the previous 3 h. Her parents denied any history of fever, rash, trauma, diarrhea or seizures. She had already been admitted to our hospital at 40 days of age because of poor feeding, vomiting, and failure to thrive. At that time, distal renal tubular acidosis was diagnosed and next-generation sequencing (NGS) identified an autosomal recessively inherited, pathogenic, homozygous mutation in exon 12 of the *ATP6V0A4 gene (NM_130841.2, c.1215C* > *G, p.F405L)*. At the last follow-up visit, three months previously, she was healthy, measuring in the 50th percentile for both height and weight. Serum bicarbonate and potassium concentrations were kept normal with potassium and bicarbonate supplementation (Table 1).

**Table 1:** Laboratory findings during the course of hospitalization

Day	Last visit^a^	Day 1^b^	Day 1 (before HD)	Day 1 (after HD)	Day 2	Day 7	Day 90	Normal range
Venous blood								
Sodium (mEq/L)	139	145	159	147	150	146	138	(139–146)
Potassium (mEq/L)	3.6	2.9	2.4	2.2	2.6	3.3	4.1	(3.87–5.4)
Anion gap (mmol/L)	12	15.6	20	12	11	10	12	
BUN (mg/dl)	10	10	7	5	8	10	9	(9–22)
Creatinine (mg/dl)	0.38	0.64	0.63	0.28	0.39	0.24	0.36	(0.2–0.45)
Glucose (mg/dl)	86	120	100	110	140	79	72	(65–100)
AST (U/L)	42	31			35	48		(27–49)
ALT (U/L)	24	18			17	107		(15–35)
Phosphorus (mg/dl)	4.4	6.1	3.1	1.3	2.0		5.3	(4.4–6.2)
CRP (mg/dl)		3	9			3		(< 5)
Ammonia (µmol/l)		515	616	130	93	55		(19–50)
Uric acid (mg/dl)	5.4	15.9	17.7	2.0	2.6	4.5	4.6	(2.2–5.6)
pH	7.39	7.21	7.31	7.63	7.45	7.42	7.38	(7.35–7.45)
pCO_2_	33.8	16	17.8	20	29	34	36.6	(32–48)
aHCO_3_	22.4	6.4	10.8	25	22	22	21.5	(22–26)
Lactate (mmol/L)	2.5	5.9	5	2	2.9	2.7	2.0	(0.63–2.44)
Urine								
pH	7.5	7.0				7.5	9.0	(5–7.5)

*BUN* blood urea nitrogen; *AST* Aspartate aminotransferase; *ALT* alanine aminotransferase; *CRP* C-reactive protein; *HD* hemodialysis

^a^Last visit is two months ago

^b^Day 1 is the initial day of hospital admission before the start of the treatment

At the time of admission to the pediatric intensive care unit, she was lethargic and severely dehydrated, her Glasgow Coma Scale was 9 (E2,M5,V2). Her body weight was 15 kg (40–50th percentile), temperature 37.0 °C, heart rate 146/min, blood pressure 70/40 mmHg, and respiratory rate 56/min. Despite dyspnea, oxygen saturation was 94%, with no cyanosis. The breathing pattern was compatible with Kussmaul breathing. Additionally, hyperreflective deep tendon reflexes were identified, combined with a dystonic response characterized by involuntary muscle contractions in the extremities. Other physical examination findings were unremarkable.

Laboratory investigations showed blood urea nitrogen 10 mg/dl, creatinine 0.64 mg/dl, calcium 9.7 mg/dl, sodium 144 mEq/L, potassium 2.9 mEq/L, uric acid 15.9 mg/dl, chloride 122 mEq/L. Severe metabolic acidosis was detected on venous blood gas analysis (pH: 7.21 and bicarbonate 6.4 mmol/l) with an increased anion gap (15.6 mmol/l) and increased serum lactate concentrations (5.9 mmol/l, Table 1). Hepatic enzymes, serum albumin, bilirubin, partial thromboplastin time, and activated partial thromboplastin time results were within the normal ranges. While blood glucose, lactate, pyruvate, the lactate/pyruvate ratio, beta-hydroxybutyrate, acetoacetate and the beta-hydroxybutyrate/acetoacetate ratio were normal, serum ammonia concentration was markedly elevated at 515 mmol/L (normal range: 19–50 mmol/L). There were no abnormalities in plasma quantitative amino acids and acylcarnitine analysis. The organic acids in the urine were within normal limits. Urinalysis revealed a pH of 7.0 with negative glucose and protein, 3 + ketones, and normal microscopic findings. Cerebrospinal fluid examination and cerebral computed tomography were normal. Electroencephalogram (EEG) revealed diffuse encephalopathy. Blood, urine, and cerebrospinal fluid cultures were sterile.

Initial treatment consisted of three 20 ml/kg boluses of normal saline. Subsequently, the patient received infusions of sodium bicarbonate (5 mEq/kg) and glucose (10 mg/kg/min), along with potassium supplementation (100 mEq in 1250 cc maintenance fluid) over six hours. Thus, the patient received 0.27 mEq/kg/hour (a total of 25 mEq) of potassium and 0.83 mEq/kg/hour (a total of 75 mEq) of bicarbonate intravenously during this period. Although this treatment normalized blood pressure, there was refractory metabolic acidosis. Moreover, serum ammonia levels increased to 616 mmol/L after six hours, despite the administration of alkaline therapy, sodium benzoate (250 mg/kg), and carglumic acid (50 mg/kg) during the six-hour management period. She was still encephalopathic and verbally unresponsive to painful stimulation on physical examination. Therefore, a 4-h hemodialysis session was performed. This treatment provided significant improvement in the metabolic acidosis and neurological symptoms, and a rapid decline in hyperammonemia. On the 3rd day of hospitalization, parenteral supportive therapies were gradually replaced by oral potassium citrate and sodium bicarbonate. The findings of encephalopathy improved on follow-up EEG. On day 7, cranial magnetic resonance imaging was performed showing normal results. At the follow-up visit after 3 months of hospitalization, she was in good clinical condition with normal neurologic examination and laboratory findings (Table 1).

## Discussion

In this study, we present a pediatric case with hyperammonemic encephalopathy which occurred due to an episode of metabolic decompensation characterized by repetitive vomiting. To our knowledge, the patient had the highest ever reported serum ammonia level in the context of pediatric distal renal tubular acidosis. Her medical condition required a session of hemodialysis which resulted in rapid clinical improvement.

The relationship between hyperammonemia and distal renal tubular acidosis was first reported by Miller et al. in a 47-day-old female patient clinically diagnosed with distal renal tubular acidosis [3]. This association has been intermittently documented in subsequent reports [4–8]. In distal renal tubular acidosis, impaired distal acidification occurs due to impaired excretion of ammonium (NH4 +) under the stimulus of systemic acidemia. However, hyperammonemia is rarely associated with distal renal tubular acidosis. Ammonia (NH_3_) and NH_4_^+^ are crucial elements in maintaining acid–base balance. Most NH_4_^+^ is synthesized in the proximal tubules before moving into the interstitium in the loop of Henle. There, it is converted into NH_3_ and accumulates in the medullary interstitium. At this point, NH_3_ enters the acidified distal tubule and medullary collecting ducts, where it acts as a buffer. Chronic metabolic acidosis and hypokalemia both stimulate renal ammoniagenesis. Elevated ammonia production, combined with impaired ammonium excretion caused by distal renal tubular acidosis, leads to the absence of 'diffusion trapping' as NH4 + in the urine. This results in the rediffusion of NH₃ back into the renal medullary interstitium, leading to an accumulation of ammonia in the bloodstream [9]. When the NH_3_ level exceeds > 100 µmol/L, neurologic findings can be seen and if > 500 µmol/L, coma and convulsions may occur. Prolongation of severe hyperammonemia may result in irreversible neurotoxicity and cerebral necrosis [10].

Hyperammonemia may more commonly occur in association with urea cycle disorders, fatty acid oxidation disorders and some organic acidurias, and can be temporary in neonates [11]. In our patient, the level of glucose, lactate, pyruvate and amino acids in the blood were all within the normal range. In addition, the patient’s urinary ketones, amino acids, and organic acids were all found to be at normal levels, which enabled us to exclude the diagnosis of a metabolic disorder. An underlying hepatic disease and Reye syndrome were also ruled out based on normal serum albumin, bilirubin, coagulation parameters and liver enzymes.

Systematic reviews and pediatric case reports reported hyperammonemia in distal renal tubular acidosis patients more commonly with *ATP6V0A4* or occasionally *ATP6V1B1* gene mutations [5, 7, 8]. In previously reported cases, the highest ammonia level was 248 mmol/L. Hypokalemia and hypobicarbonatemia were frequently observed concomitantly and were negatively correlated with hyperammonemia. Neurologic findings were usually absent, except for mild confusion noted in some cases. The ammonia level rapidly normalized with alkaline therapy and when potassium levels reached high-normal levels. None of those patients required dialysis [3–8]. Our patient also had an underlying *ATP6V0A4* gene mutation. However, complaints of repetitive vomiting and poor feeding for a few days resulted in metabolic decompensation, with severely elevated serum ammonia level above 500 µmol/L at admission, accompanied by encephalopathy.

Despite the efforts to normalize metabolic acidosis and hypokalemia via the use of alkaline therapy, potassium supplementation, and non-kidney replacement therapy consisting of nitrogen scavenger agents, ammonia level further increased in our patient and altered consciousness persisted after 6 h of the medical treatment. A recent guideline recommends employing hemodialysis for patients with ammonia levels > 500 mmol/L with clinical signs of severe encephalopathy or those with lower levels if an insufficient clinical response is achieved after 4 h of appropriate medical treatment. It is also stated that the main determinants of neurological outcome are plasma ammonia levels and duration of hyperammonemic coma [12]. Therefore, we believe that initiating dialysis in patients with severely elevated ammonia levels and keeping the duration of hyperammonemia as short as possible should be important management strategies. Considering this literature information, we implemented hemodialysis in our patient.

In the present case, intermittent hemodialysis was preferred over continuous kidney replacement therapy as the dialysis modality. It is reported that within 3–4 h of a treatment session, a 75% decline in blood ammonia levels can be achieved via intermittent hemodialysis [12]. Continuous dialysis modalities are also known to be effective in treating hyperammonemia, whereas continuous venovenous hemodialysis (CVVH) offers relatively better ammonia clearance than continuous venovenous hemodiafiltration (CVVHD). On the other hand, previous pediatric studies showed that intermittent hemodialysis provided a significantly more rapid reduction in serum ammonia levels than CVVHD [10, 13, 14]. Another point was that our patient also had severe metabolic acidosis due to underlying uncompensated distal renal tubular acidosis. It is well-known that severe metabolic acidosis can be more efficiently managed with intermittent hemodialysis compared to continuous kidney replacement therapy options given that the patient is hemodynamically stable [15]. In summary, considering all the above literature information, intermittent hemodialysis was the treatment of choice in our patient. Although rebound hyperammonemia can be seen more often with intermittent hemodialysis, and may necessitate repeated sessions [12], serum ammonia levels rapidly decreased and no rebound increase was detected in our patient. Furthermore, no long-term neurologic sequelae were observed.

The association between distal renal tubular acidosis and hyperammonemia has been demonstrated not only in pediatric cases but also in adult patients. A 69-year-old woman was hospitalized with a reduced level of consciousness, fatigue, generalized weakness, anorexia, and two weeks of polyuria. She was diagnosed with distal renal tubular acidosis which developed secondary to primary hyperparathyroidism and hyperammonemia. Following alkalizing treatment, her ammonia level of 278 µmol/L decreased to normal range, and she was discharged without any residual neurological deficits [16]. Another reported patient was a 72-year-old Japanese man, who had been taking omeprazole for 11 years that was admitted to the hospital due to loss of consciousness and muscle weakness. He was diagnosed with omeprazole-induced hypomagnesemia, leading to possible distal renal tubular acidosis with hypokalemia, hypocalcemia, hyperlactacidemia, and hyperammonemia (ammonia level of 174 µmol/L). After correcting his serum electrolytes and administering alkaline therapy, the patient's muscle weakness and consciousness improved, and his ammonia level normalized. There was no evidence of neurological impairment upon discharge [17].

## Conclusion

This case report highlights the importance of early recognition and rapid management of risk factors that may trigger metabolic decompensation characterized by deep metabolic acidosis, hypokalemia and subsequent hyperammonemia in distal renal tubular acidosis patients. As ammonia levels can reach critical values that may necessitate hemodialysis despite intensive medical therapy, we suggest that it should be routinely measured in children with distal renal tubular acidosis, particularly during follow-up when the disease is associated with repetitive vomiting or signs of encephalopathy, as in our patient.

## Data Availability

Data sharing is not applicable as no new data were generated or analyzed in support of this research, and the article describes a case report.
